# The Evolution of Spinnable Cotton Fiber Entailed Prolonged Development and a Novel Metabolism

**DOI:** 10.1371/journal.pgen.0040025

**Published:** 2008-02-01

**Authors:** Ran Hovav, Joshua A Udall, Bhupendra Chaudhary, Einat Hovav, Lex Flagel, Guanjing Hu, Jonathan F Wendel

**Affiliations:** Department of Ecology, Evolution and Organismal Biology, Iowa State University, Ames, Iowa, United States of America; Washington University, United States of America

## Abstract

A central question in evolutionary biology concerns the developmental processes by which new phenotypes arise. An exceptional example of evolutionary innovation is the single-celled seed trichome in *Gossypium* (“cotton fiber”). We have used fiber development in *Gossypium* as a system to understand how morphology can rapidly evolve. Fiber has undergone considerable morphological changes between the short, tightly adherent fibers of G. longicalyx and the derived long, spinnable fibers of its closest relative, G. herbaceum, which facilitated cotton domestication. We conducted comparative gene expression profiling across a developmental time-course of fibers from *G. longicalyx* and G. herbaceum using microarrays with ∼22,000 genes. Expression changes between stages were temporally protracted in G. herbaceum relative to G. longicalyx, reflecting a prolongation of the ancestral developmental program. Gene expression and GO analyses showed that many genes involved with stress responses were upregulated early in G. longicalyx fiber development. Several candidate genes upregulated in G. herbaceum have been implicated in regulating redox levels and cell elongation processes. Three genes previously shown to modulate hydrogen peroxide levels were consistently expressed in domesticated and wild cotton species with long fibers, but expression was not detected by quantitative real time-PCR in wild species with short fibers. Hydrogen peroxide is important for cell elongation, but at high concentrations it becomes toxic, activating stress processes that may lead to early onset of secondary cell wall synthesis and the end of cell elongation. These observations suggest that the evolution of long spinnable fibers in cotton was accompanied by novel expression of genes assisting in the regulation of reactive oxygen species levels. Our data suggest a model for the evolutionary origin of a novel morphology through differential gene regulation causing prolongation of an ancestral developmental program.

## Author Summary

Human domestication of plants has resulted in dramatic changes in mature structures, often over relatively short time frames. The availability of both wild and domesticated forms of domesticated species provides an opportunity to understand the genetic and developmental steps involved in domestication, thereby providing a model of how the evolutionary process shapes phenotypes. Here we use a comparative approach to explore the evolutionary innovations leading to modern cotton fiber, which represent some of the more remarkable single-celled hairs in the plant kingdom. We used microarrays assaying approximately 22,000 genes to elucidate expression differences across a developmental time-course of fibers from *G. longicalyx*, representing wild cotton, and G. herbaceum, a cultivated species. Expression changes between stages were temporally elongated in G. herbaceum relative to G. longicalyx, showing that domestication involved a prolongation of an ancestral developmental program. These data and quantitative real time-PCR experiments showed that long, spinnable fiber is associated with a number of genes implicated in regulating redox levels and cell elongation processes, suggesting that the evolution of spinnable cotton fiber entailed a novel metabolic regulatory program

## Introduction

One of the central questions in evolutionary biology concerns the developmental and genetic processes by which new phenotypes arise. The recent merger of genomic technologies with phylogenetics has generated important insights into the evolution of developmental transformations in maize [1,2], rice [3,4] and other taxa, e.g., [5,6]. These studies demonstrate that morphological change in complex organs can often be initiated by relatively few mutations, most often in regulatory regions [7], although the genetic underpinnings of most evolutionary change remains unknown. An exceptional example of evolutionary innovation involving a single-celled structure is the cotton seed trichome, present in all 50 species in the genus *Gossypium* and colloquially termed “cotton fiber” in the domesticated species. On the day of anthesis (flower opening), approximately one in four cells of the ovular epidermis has already been fated to become a trichome, initially appearing as a spherical protrusion and subsequently elongating through stages of primary wall synthesis, secondary wall synthesis, maturation and cell death. Representing one of most distinct single cell types in the plant kingdom, cotton fibers may attain a final length of 6 cm in some cultivars, with a length/width ratio of more than 2000 [8]. A single cotton ovary contains ∼500,000 elongating cells representing a single cell type.

**Figure 1:**
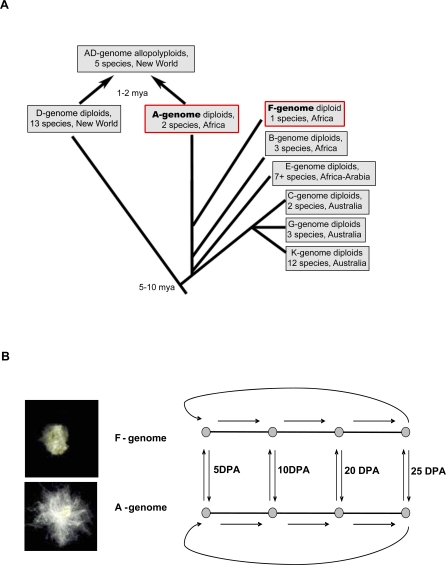
Evolutionary History of Cotton in Relation to the Current Study (A) Evolutionary history of diploid and tetraploid *Gossypium* species groups (*n* = 13 and 26, respectively), as inferred from multiple molecular datasets [11,13], indicating sister-taxon relationship between the F- and A- genomes. F-, G. longicalyx (short fibers); A-, G. herbaceum (long fibers). (B) Microarray experimental loop-design for detecting gene expression changes during the development of A- and F-genome fibers. Arrows denote dyes that were used in each of the hybridizations (tails, Cy3; arrow heads, Cy5).

The long, strong and fine fibers of modern cotton cultivars were wrought through a long history of both natural and human-mediated selection [9–11]. Following its origin about 10 MYA [12,13], *Gossypium* diversified into approximately 50 species in the warmer, arid to semi-arid regions of both hemispheres. This radiation was accompanied by cytogenetic differentiation, which now is reflected in the recognition of eight, monophyletic “genome groups” (A to G and K) (Figure 1A). Remarkably, four wild *Gossypium* species were independently domesticated by aboriginal domesticators ∼5000 years ago, or more, and transformed into fiber and seed-oil plants [10,14]. Two of these (G. arboreum and G. herbaceum) are A-genome diploids from the Old World, while the other two (G. hirsutum and G. barbadense) are AD-genome allotetraploids from the New World.

In contrast to the cultivated diploids and tetraploids, wild diploid species have short (mostly <5mm), coarse and tightly adherent trichomes that would not be recognized as “cotton” by a casual observer. The only exception is the wild form of G. herbaceum (G. herbaceum subsp. *africanum*), which has sparse but elongated and spinnable fiber. Both wild and cultivated cottons produce fiber on the seed coat, but there are striking morphological and structural differences between these fibers, the most obvious of which is their size. Some species (e.g., *G.thurberi, G. trilobum, G. davidsonii,* and *G. klotzschianum*) do not possess obvious seed hairs, but they actually are present as developmentally repressed structures not visible to the unaided eye [15]. The duration of the elongation phase and the timing of onset of secondary wall synthesis appear to be key determinants of the final length of the fiber in both wild and cultivated plants [15–18]. This trait of prolonged elongation was passed on to the allopolyploids, which in turn was a key component of their eventual domestication [15].

When viewed in a phylogenetic context (Figure 1A), the origin of spinnable fibers is diagnosed to have occurred once in the history of *Gossypium*, following divergence of the A-genome and F-genome clades. This “pre-adapted” A-genome ancestor later contributed this genome and its propensity for the development of elongated cotton fibers to the allotetraploid cottons that colonized and diversified in the New World, ultimately giving rise to the modern, annualized forms of G. hirsutum that account for >90% of contemporary world cotton commerce.

To gain insight into the genetic factors that led to the evolution of long, spinnable cotton fibers, we performed global gene expression analysis, comparing the A-genome taxon G. herbaceum to its closest wild relative, G. longicalyx. The latter species was described relatively recently, following its discovery in eastern Africa [19], and is cladistically resolved as the sister taxon to the A-genome in molecular phylogenetic analyses of both plastid and nuclear gene sequences (13) (Figure 1A). We used a newly designed long oligonucleotide microarray and ovular trichome isolation procedures to analyze gene expression over a time-course of trichome development in both species. This analysis revealed major differences in genes related to stress responses and cell elongation, as well as a prolonged developmental profile in wild G. herbaceum. We suggest that the evolution of spinnable fiber was accompanied by prolongation of an ancestral developmental program, mediated through avoidance of stress-like processes in the developing fiber cells. This suggests that the evolutionary origin of a novel cell phenotype was facilitated by hypermorphosis.

## Results

### The A-Genome Shows a Prolonged Fiber-Cell Developmental Program

**Figure 2:**
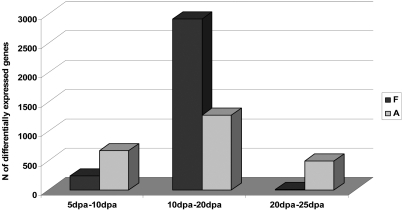
Summary of the Number of Genes Differentially Expressed between Adjacent Time-Points during Fiber Development (FDR < 0.05)

We used an experimental loop-design system to compare mRNA expression levels in developing fiber-cells derived from A- and F-genome cotton species (Figure 1B). RNAs isolated from four developmental time-points, 5, 10, 20 and 25 days post-anthesis (DPA), were amplified and hybridized to cotton oligonucleotide microarrays containing 22,827 probes derived from deep EST sampling of diverse tissues and organs [20]. A summary of the number of genes differentially expressed between adjacent time-points during fiber development in both species is presented in Figure 2. Within each genome, many genes were found to be differentially expressed during fiber development (FDR < 0.05), but the distribution of the number of genes differentially expressed between adjacent time points was different for the two species. In G. herbaceum, the distribution was relatively even throughout the developmental stages studied (notice that the interval 10–20 DPA is twice the duration of 5–10 and 20–25 DPA), whereas in the F-genome species, G. longicalyx, ∼80% of significant expression changes occurred between the two adjacent time points 10 and 20 DPA. Also, in the transition from 20 to 25 DPA only 4 genes were differentially expressed in G. longicalyx, in comparison to 493 genes that were differentially expressed during this same interval in the A-genome species G. herbaceum. Because longer, spinnable fiber is phylogenetically derived [15], these results indicate that the fiber-cell developmental program in the A-genome has become prolonged during its evolution.

### F-Genome Fiber Development Is Linked with Early Expression of Stress-Related Genes

To better appreciate changes in global expression during fiber development and evolution, we tracked differences in expression between A- and F-genomes for all genes at all developmental stages. For each comparison upregulated and downregulated genes were tabulated, from which we derived categories of statistically overrepresented biological processes (Table S1). As expected from an inter-specific comparison, hundreds of genes were found that were differentially expressed between fibers from A- and F-genome plants. Inspection of the gene lists revealed that, among genes which previously had been described as regulating fiber elongation [21], some did indeed show significant expression differences, while others did not. An example of the developmental expression patterns for 5 differentially expressed and 5 non-differentially expressed, well-described genes, between F- and A- genomes, is provided in Figure S1.

A major difference in the developmental programs of fiber cells in the two taxa was revealed by GO family representation analyses (Table S1). Most noteworthy is the observation that at the beginning of fiber development in F-genome fibers, many genes involved with stress-response processes were highly upregulated. Comparison of statistically overrepresented biological processes at 5 DPA, for example, showed that in A-genome fibers, processes important for elongation, such as “respiration”, “energy” and “ribosome biogenesis” are overrepresented (Table 1). At the same time-point, however, genes upregulated in F-genome fibers belong to biological processes such as “response to stress”, “transcription regulatory activity” and “flavonoid biosynthesis”. Moreover, analyzing the 60 most-differentially expressed genes at 5 DPA, representing the top 2% of the upregulated genes in the F-genome in comparison to the A-genome, showed that more than a third were related to “response to stress” processes (Table S2). The expression pattern of some of these highly upregulated “stress response” genes in developing fiber-cells of the F-genome in comparison to the A-genome are presented in Figure S2.

**Table 1:**
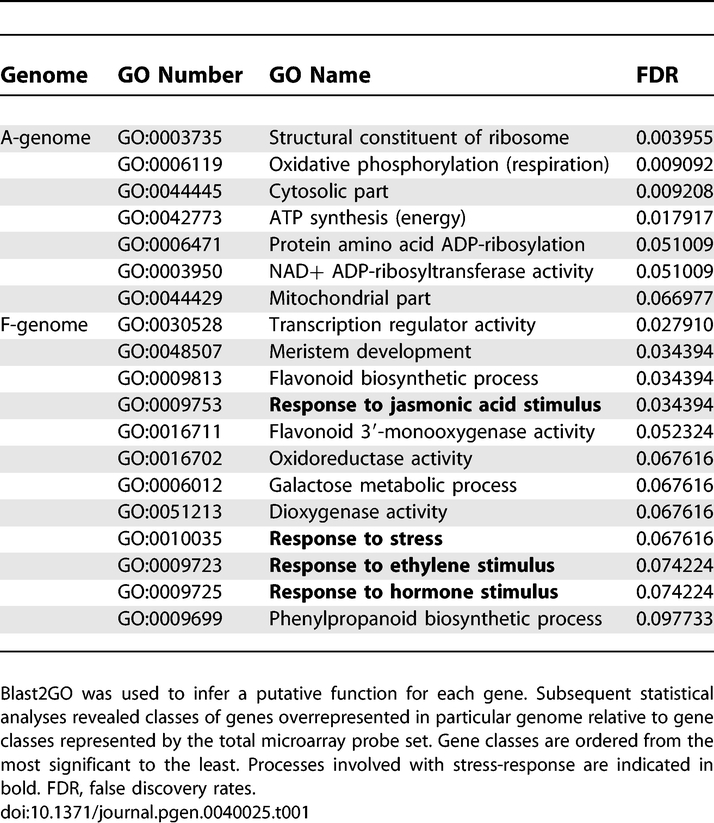
Biological Processes Overrepresented in A- and F- Genomes at 5 DPA

### Overexpressed Genes in A-Genome Fibers Are Associated with H_2_O_2_ and ROS Regulation

A possibility that emerged from differences in gene expression between A-genome and F-genome fibers was that high levels of H_2_O_2_ and other reactive oxygen species (ROS) may be responsible for the stress-like processes evident in F-genome fibers early in fiber development. Hydrogen peroxide has previously been shown to be important for cell elongation, as it is required for cell wall loosening and expansion [22–25]. At high concentration, however, H_2_O_2_ becomes toxic, leading to stress processes that may lead to early onset of secondary cell wall synthesis and the end of cell elongation [22,26]. These observations suggest the hypothesis that the evolution of long spinnable A-genome fibers was accompanied by novel expression of genes that assist in regulating H_2_O_2_ and other ROS levels.

To evaluate this possibility, we examined the microarray data for genes that may control ROS and cell stress in elongating cells (Table 2). Three genes shown in other systems to regulate H_2_O_2_ levels by functional or regulatory means were investigated further. *GAST1-like* is a member of the gibberellin-induced, cysteine-rich protein family previously shown to be induced by H_2_O_2_. In transgenic *Petunia*, suppression of *GAST1-like* homologs inhibits elongation, whereas upregulation stimulates H_2_O_2_ scavenging, perhaps via redox-active cysteines, and cell elongation [27]. *GAST1-like* was previously shown to be expressed mainly in cotton fibers [21] (Figure S3 **)**. *Cop1/BONZAI* is part of a calcium-dependent, membrane-binding protein family, shown to promote growth and development in addition to repression of cell death by inactivation of stress-promoting R-genes [28]. *Pex1* is a gene that encodes a protein important for the biogenesis of the peroxisome, an organelle that rids the cell of toxic substances such as H_2_O_2_ [29]. Both *Cop1/BONZAI* and Pex1 genes are only detected in fiber-specific EST libraries, out of more than 30 EST libraries that exist for cotton from a diverse set of tissues and organs (http://cottonevolution.info/).

**Table 2:**
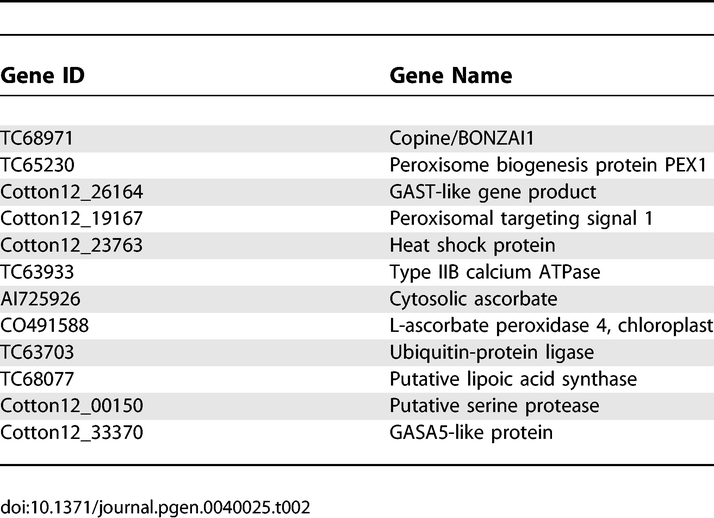
Candidate Genes Upregulated in A-Genome Controlling ROS, H_2_O_2_, and Cell Stress in Elongating Cells

**Figure 3:**
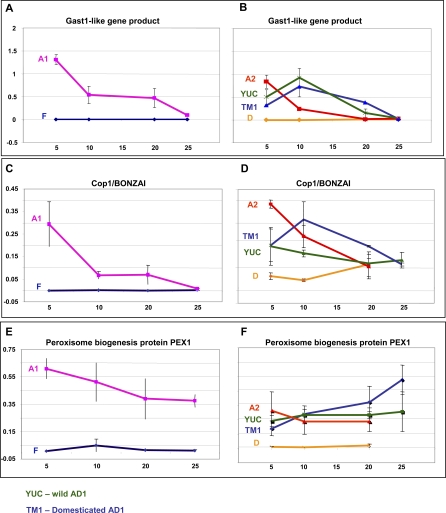
Quantitative Real-Time PCR Analyses of Three Candidate Genes Controlling H_2_O_2_ Levels *Gossypium* species investigated were G. herbaceum (A1), G. longicalyx (F) (left panel); G. hirsutum var TM1 (TM1), G. hirsutum var *yucatanense* (YUC), G. arboreum (A2), and G. raimondii (D5) (right panel). Each point on the graph represents the mean of three biological replications.

For each of these three genes, we conducted real-time PCR using elongating and non-elongating fibers from several additional cotton accessions and species with either short or long fiber. As shown in Figure 3, *GAST1-like*, *Cop1/BONZAI* and *Pex1* were highly upregulated in A-genome relative to F-genome fibers. Paralleling this result, all three genes were expressed in additional taxa having long fibers (G. arboreum, and both cultivated and wild forms of G. hirsutum), at the beginning of fiber-cell development, but were either undetected or were only weakly expressed in species with short fibers (G. raimondii (D clade)*,* in addition to the F-genome species *G. longicalyx*). This consistent association between fiber development and gene expression across divergent clades suggests a functional association.

## Discussion

Transcription profiling of cotton using microarrays has been the subject of several recent studies, either using ovular tissue with fibers attached, or the fibers themselves [21,30–33]. These studies simultaneously evaluate mRNA expression levels for thousands of genes, providing a powerful tool for analyzing biological processes important to cotton fiber differentiation and development. One conclusion is that the transcriptome of cotton fibers is extraordinarily complex [21], involving thousands of genes that vary in expression levels through the stages of cellular initiation, primary wall synthesis, secondary wall deposition, maturation, and death. Here we provide the first comparative evolutionary analysis of fiber cell development, focusing on the initial phylogenetic steps implicated in the natural transformation of epidermal seed trichomes into long, spinnable fibers prior to and during human domestication. In this regard, the accession we used in this study is a domesticated form of G. herbaceum. Thus, our study reflects the evolution of either species-level differences, human selection (domestication), or both. The above-described qPCR analysis, however, was performed using other domesticated and wild species, showing that for this set of genes the changes occurred prior to domestication. In addition to its relevance to the evolutionary transitions in morphology and to cotton crop improvement, to our knowledge this study is the first to characterize the evolutionary, developmental genomics of a single cell type in any eukaryotic organism.

**Figure 4:**
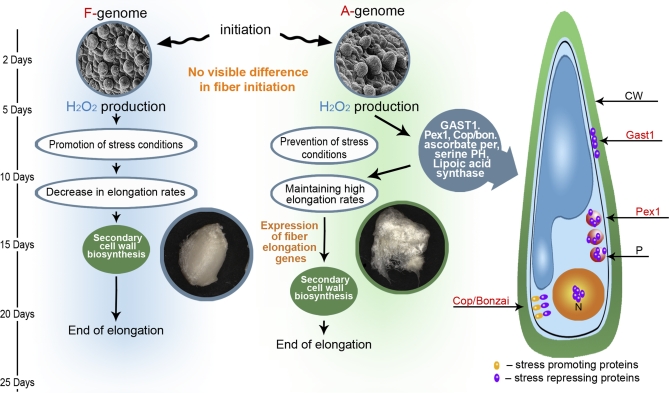
An Evolutionary and Development Model Describing Processes That Lead to the Formation of Spinnable Fiber

Our proposed model describing the developmental and evolutionary processes that led to the formation of spinnable fiber is presented in Figure 4. Previous ultrastructural characterization of various developing cotton fibers, including those of F- and A- genome species, demonstrate that the earliest stages of fiber initiation and development are phenotypically similar for species with either short or long fibers [15]. At 2 DPA, for example, fiber cells appear as the same spherical protrusion both in F- and A- genomes. This stage is followed by a period of rapid cell elongation, a process known to involve cell-wall relaxation, which itself has been shown to require non-enzymatic reactions mediated by H_2_O_2_ and other ROS that cleave polysaccharides [22–25]. Therefore, H_2_O_2_, produced enzymatically by oxidation reactions, is a necessary molecule for cell elongation. Higher levels of H_2_O_2_, however, may halt elongation through apparent stimulation of cell wall stiffening [22,26], and can even promote programmed cell death or necrosis. Accordingly, Cosgrove [34] has suggested that fine regulation of steady-state levels of ROS are essential for proper cell elongation.

Our model suggests that the curtailed developmental duration of the F-genome, relative to the A-genome, is caused by an insufficient control of cellular H_2_O_2_ and other ROS levels, eventually arresting elongation and leading to an induction of secondary cell wall synthesis. In cotton, H_2_O_2_ has been suggested to function as a developmental signal in the differentiation of secondary walls in cultivated G. hirsutum fibers, evidenced by the fact that inhibition of H_2_O_2_ production or scavenging existing H_2_O_2_ from the system prevents cell wall differentiation [35]. Similarly, exogenous addition of H_2_O_2_ prematurely promotes secondary cell wall formation in young fibers [35]. Our data are consistent with this interpretation and with earlier secondary cell wall formation in the F-genome, as indicated by expression differences between F- and A- genomes for the cellulose synthase A1 (*CeSA1*) gene. *CeSA1* is a well-characterized gene involved in fiber secondary cell wall synthesis, and *CeSA1* RNA expression levels have been suggested as a marker for secondary wall cell synthesis [30]. In our study, *CeSA1* increases its expression earlier in the F-genome than in the A-genome (Figure S1–S1J).

Similar to the F-genome, early elongating fibers of the A-genome are exposed to increasing levels of H_2_O_2_. A-genome fibers, however, did not show increased RNA levels of stress-related genes, suggesting that this lineage evolved a metabolism to modulate ROS levels by either functional or regulatory means. At the functional level, genes controlling antioxidant levels, including ascorbate peroxidase, glutathione peroxidase and lipolic acid synthase, are all upregulated in A-genome fibers relative to those of the F-genome. These proteins scavenge and detoxify H_2_O_2_ and other ROS. Recently, comparative proteomic analysis between regular and mutant cotton fibers showed the involvement of a cotton ascorbate peroxidase in H_2_O_2_ homeostasis during cell development [36].

An additional example of possible functional modulation of H_2_O_2_ levels is offered by the protein *GAST1*, which we studied further. *GAST1* is a cysteine-rich, gibberellin-induced gene, initially identified in tomato, which is suppressed in the GA-deficient mutant *gib-1* and for which expression coincides with stem elongation [37]. It belongs to a protein family, identified in many plant species, that is suggested to play a role in many biological processes, including cell division, cell elongation (promotion and inhibition), transition to flowering, root development, fruit development and defense (summarized in [27]). RNAi suppression of expression in one member of this family, *GIP2,* was shown to inhibit stem elongation under low-temperature conditions in transgenic *Petunia* [38]. Wigoda et al. [27] have shown that *GIP2* overexpression promotes stem and corolla elongation. In the same study they showed that *GAST2* is expressed in the cell-wall and suggested that its putative redox-active cysteines may act as antioxidants that control H_2_O_2_ levels at this site. The fact that our cotton *GAST1* has the same conserved 12 cysteine residues as other *GAST-like* proteins (data not shown), is expressed mainly in the fiber [21] (as shown in Figure S3), and is not expressed in the fuzz-like F-genome fibers and the D-genome fibers make it a promising gene for further investigation.

At the regulatory level, we explored two candidate genes further using quantitative RT-PCR and a broader sampling of species and accessions having either short or long fibers. The first is the *Cop1/BONZAI* (*Cop1*) gene. *Cop1* is a calcium-dependent, membrane-binding protein isolated from a mutant with a temperature-dependent growth defect and an enhanced disease resistance phenotype in *Arabidopsis* [39,40]. Further study revealed that *Cop1* acts as a repressor of a disease resistance (R) gene and as such it prevented programmed cell death (PCD) processes (i.e. hypersensitive response) [28]. It is unclear if the F-genome fiber is undergoing early “classical” PCD processes, indicated by the fact that “cell death processes” was not a statistically overrepresented biological process in our study. *Cop1*, however, was ranked in our microarray analysis as the single most upregulated gene in the A-genome, relative to the F-genome, among thousands of differentially expressed genes, making it as a strong candidate for controlling stress-like processes. Further quantitative RT-PCR analyses using a broader sampling of species with short and long fibers yielded comparable results (Figure 3), lending additional support to the hypothesis of a role for *Cop1*in fiber elongation.

A second regulatory gene studied further is *Pex1*. This gene is one of a cascade of peroxisome biogenesis genes that previously have been shown to be induced by H_2_O_2_ in both plant and animal cells, and have been suggested to assist in restoration of cellular redox balance in response to wounding and infection with an avirulent pathogen [29]. *Pex1* encodes a member of the AAA (ATPases associated with diverse cellular activities) superfamily of ATPases that have been suggested to mediate lipid and/or membrane addition to peroxisome membranes, facilitating peroxisome growth [41,42]. As shown in Figure 3, *Pex1* mRNA levels are strongly correlated with fiber length.

To verify the hypothesized roles of *GAST1, Cop1*, and *Pex1* genes, additional functional analyses are needed in growing fiber-cells, including *in vivo* measurements of H_2_O_2_ levels and elongation rates in developing fibers derived from F- and A-genome species. These studies comprise one focus of our ongoing efforts. In addition, hundreds of other genes were differentially expressed between A- and F-genome fibers, including many known to be involved in fiber development, suggesting that in addition to cellular redox balance, other biological processes may be involved in the evolution of spinnable fibers. Thus, additional work is necessary to reveal the nature and relative contributions of these additional processes to fiber transformation during evolution.

The results presented here add perspective to results from our previous comparative study of fiber development in wild *Gossypium* species [15], in which wild A- and F-genome species exhibited continued fiber elongation up until approximately 21 DPA. One possible reconciliation between this observation and the present study is that the timing of the period of *maximum* fiber elongation is a key developmental difference. Some species with short fibers showed a nearly linear rate of elongation over most of the growth period, whereas long-linted species exhibited more complex growth curves. Another possibility is that the most important factor in determining final fiber length is the absolute fiber elongation rate and not the relative percentage of mature length (as [15]), suggesting that the effects of H_2_O_2_ and other ROS are not qualitative, but instead are quantitative, operating via effects on *amount* of elongation.

The present study implicates several molecular mechanisms as being involved in the evolution of elongated epidermal seed trichomes, providing the foundation for later human domestication of an important crop plant. Why elongated epidermal seed hairs evolved is a matter of speculation. Perhaps fibers evolved to aid in bird dispersal, as suggested [43]. This hypothesis gains credibility from observations by J. Stewart (unpubl.) of a bird's nest in NW Puerto Rico that contained numerous seeds of feral G. hirsutum, as well as a collection of G. darwinii from a finch's nest in the Galapagos Islands (J. Wendel, unpubl.). One might also speculate that fibers serve to inhibit germination unless there is sufficient moisture to saturate the fibers; should germination occur following a light rain, there might not be sufficient water for subsequent survival of the seedling. A related possibility is that seed hairs function as “biological incubators” to facilitate germination only when ecological conditions are appropriate, by recruiting particular microbial communities under appropriate moisture regimes. Irrespective of the veracity of these speculations, one outcome is that these processes set the stage for human domestication millions of years later.

We show that a major heterochronic change included prolongation of an ancestral developmental program, and coincidently, this change pre-adapted the derivative cell type for human domestication. At least in part it appears that avoidance or delay of stress-like processes may underlie the increased elongation in A-genome fiber development compared to F-genome fiber, in conjunction with an increased ability to modulate cellular redox balance in the growing cell. These evolutionary processes, occurring as they did perhaps several million years ago [9,10], may be interpreted as the key events responsible for the domestication of one of the world's most important crop plants.

## Materials and Methods

### Plant material and RNA preparation.

Plants from Gossypium herbaceum (A1), G. longicalyx (F), G. hirsutum var TM1 (AD1), G. hirsutum var *yucatanense* (wild AD1), G. arboreum (A2) and G. raimondii (D5) were grown in three separate replicates of 4–8 plants in the Horticulture Greenhouses at Iowa State University. For each replicate, ovules were excised, immediately frozen in liquid nitrogen, and stored in −80 °C. At each developmental time point, fibers were isolated from ovules using a liquid nitrogen/glass bead shearing approach [21]. Initially, ovules were visually inspected for cell damage and the fibers were inspected for contaminating tissue. Subsequent RNA extractions were performed using a hot borate method [44]. RNA quality was confirmed on a BioAnalyzer (Agilent, Palo Alto, CA).

### RNA amplification, labeling, and microarray hybridizations.

For microarray analyses, an indirect labeling procedure of amplified aminoallyl a-RNA was used as described [21]. Two dyes, Cy3 and Cy5, were coupled to 8 μg aliquots of aRNA using the Post-Labelling Aminoallyl-aRNA CyDye reactive dyes (Amersham Biosciences). A newly designed cotton long-oligonucleotide microarray containing over 22,827 probes derived from deep EST sampling of diverse tissues and organs [20] was used. All hybridizations, slide scanning and normalizations were performed as described previously [21].

### Experimental design and statistical analysis.

A balanced developmental loop design for microarray analysis was performed (Figure 1B). For G. herbaceum (A1) and G. longicalyx (F), four fiber developmental time-points, 5, 10, 20 and 25 DPA were sampled. Within each species, hybridizations were performed between each pair of consecutive developmental stages by labeling one with Cy5 and the other with Cy3, and by closing the loop with a comparison of 25 and 5 *DPA*. In addition, 2 hybridizations were done between species at each time-point, using a dye swap for each pair. With three biological replications and 16 slides each, we generated gene expression data from a total of 48 microarrays. Statistical analyses were performed using R and SAS statistical software (code available upon request).

Log transformed, median-normalized values of the 22,827 genes were examined for expression differences between each fiber developmental stage within and between species. We considered a standard mixed linear model for the data for each gene as:

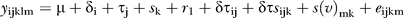
where *y*_ijklm_ denotes the normalized log-scale signal intensity for genotype *i*, time-point *j*, biological replication *k*, dye *l* and slide *m*; μ denotes an intercept parameter; δ_i_ denotes the fixed effect of genotype *i*; τ_j_ denotes the fixed effect of time-point *j*; *s*_k_ denotes the random effect of replication *k*; *r*_l_ denotes the fixed effect of dye *l*; δτ_ij_ denotes the fixed effect of the interaction between genotype *i* and time-point *j*; δτ*s*_ijk_ denotes the random effect of the interaction between genotype *i*, time-point *j* and replication *k*; *s*(*v*)_mk_ denotes the effect of the slide m inside replication *k*; *e*_ijkl_ denotes a random error term intended to capture all other sources of variability. Contrasts for differential expression between genotypes, time points and the interaction genotype *x* time-points were conducted using this model. For each gene, differences were calculated using the following pair-wise contrasts:

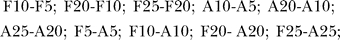
where letters denote genotype (F- or A-genome) and numbers denote developmental time-point (5, 10, 20, or 25 DPA).

The 22,827 *p*-values from each comparison were converted to q-values using the method of Storey and Tibshirani [45]. These q-values were used to identify the number of differentially expressed genes for a given comparison when controlling the false discovery rate (FDR) at various levels.

Blast2GO (http://www.blast2go.de/) was used to identify biochemical pathways involved in a given comparison and to calculate the statistical significance of each pathway. Blast2GO includes the Gossip package [46] for statistical assessment of annotation differences between two sets of sequences, using Fisher's exact test for each GO term. FDR controlled *p*-values (FDR < 0.05) were used for the assessment of differentially significant metabolic pathways.

### Quantitative RT-PCR analyses.

Amplified aRNA was used as a template for first strand cDNA synthesis with the Super-script II pre-amplification system reverse transcriptase kit (Gibco BRL Life Technologies) at 42 °C according to the supplier's instructions. Specific primers with amplicons for quantitative PCR were designed based on the sequence derived from the EST sequence corresponding to the candidate and reference genes (Table S3). We used the RNA helicase gene (Q9ZS12) as the reference gene. RNA helicase gene was found to be equally expressed in different developing fibers as well in other plant tissues [21]. cDNA was used as the template for quantitative PCR amplification using the GeneAmp 5700 Sequence Detection System (PE Biosystems) with SYBR Green Master Mix containing AmpliTaq Gold, according to the manufacturer's instructions (PE Biosystems). Standards containing logarithmically increasing known levels of cDNA were run with each set of primers for normalization. All real-time PCR products were confirmed by sequencing.

## Supporting Information

Figure S1 Expression Patterns of Previously Well-Characterized Genes Involved in Fiber Development [21] The graphs show the difference in LS means between the A- and F- genome fibers during development.
(A–E) Genes associated with fiber development that do not show a difference in expression between F- and A-genomes.
(F–J) Genes associated with fiber development that show significant difference in expression between F- and A-genomes. Triangles, A-genome; squares, F-genome.
(3.7 MB TIF)

Figure S2 Expression Patterns of Six Highly Upregulated “Stress-Response” Genes in Developing Fibers of the F-Genome in Comparison to the A-Genome Values represent the log-normalized LSmeans of three biological replications. Squares denote the F-genome and triangles the A-genome. The genes presented are: (A) (Q84YH9) Polyphenol oxidase. (B) (Q84QI6) Auxin response factor. (C) (O22616) Ornithine decarboxylase. (D) (Q9M662) Cell death associated protein. (E) (Q9ZVR6) Jasmonic acid induced lectin. (F) (Q84YB7) cystein proteinase RD19a. For more details about the selected genes, please refer to Table S2.
(1.6 MB TIF)

Figure S3 Difference in Expression of the *Gast1*-Like Gene Product The comparison is between populations of developing cotton fiber cells and a genetically complex reference sample derived from six different cotton organs, showing that the *Gast1*-like gene is fiber-specific. Data are from Hovav et al. [21].
(1.5 MB TIF)

Table S1 Number and Overrepresented Classes of Upregulated and Downregulated Genes Blast2GO was used to infer a putative function for each gene. Subsequent statistical analyses revealed classes of genes overrepresented in particular time-point/species relative to gene classes represented by the total microarray probe set. Gene classes are ordered from the most significant to the least.
(39 KB XLS)

Table S2 List of 2% Upregulated Genes in the F-Genome in Comparison to the A-Genome at 5 DPA The table shows the genes ID and the first blast hit for each gene. Genes associated with stress-like processes are indicated in red. For these genes a web-based URL example is attached.
(26 KB XLS)

Table S3 Primers Used in This Study for Real-Time PCR (30 KB XLS)
