# Supplementary material for: The Evolution of Spinnable Cotton Fiber Entailed Prolonged Development and a Novel Metabolism
Source: PLoS Genet. 2008 Feb 1;4(2):e25. doi: 10.1371/journal.pgen.0040025 (PMC2222923; doi:10.1371/journal.pgen.0040025)
Supplement: Supplementary file 1 [file pgen.0040025.st003.doc]

Supplemental Table 3

| **Name** | **Sequence (5’ to 3’)** | **Amplicon (bp)** | **Gene product** |
| --- | --- | --- | --- |
| GAST F | TCCATAAGTAAAAAGACCGACTG | 95 | Gast-like protein |
| GASTB R | CAAGGGCAGGCTTCATAGTTGC | 95 | Gast-like protein |
| Copine F 2 | TTCCTTTCACGCCTTGAGTTTGGC | 159 | Bon1 |
| Copine R2 | CAATGCAACCATAGCCAGCAGACA | 159 | Bon1 |
| PEX1 F | TTCCTTGCCAAGAAGCTGAAGACTGC | 140 | Pex1 |
| PEX1 R | CAACGTGTTAACTTCCGTCTCTGC | 140 | Pex1 |
| reference-F | AGCCTGATCGATATGTGGAGGGAT | 180 | (Q9ZS12) RNA helicase |
| reference-R | TCAGGAAGGTTTGGCCATCTTGGA | 180 | (Q9ZS12) RNA helicase |
